# Detecting Single Microwave Photons with NV Centers in Diamond

**DOI:** 10.3390/ma16083274

**Published:** 2023-04-21

**Authors:** Olivia Woodman, Abdolreza Pasharavesh, Christopher Wilson, Michal Bajcsy

**Affiliations:** 1 Institute for Quantum Computing, University of Waterloo, Waterloo, ON N2L 3G1, Canada; 2 Department of Physics and Astronomy, University of Waterloo, Waterloo, ON N2L 3G1, Canada; 3 Department of Electrical and Computer Engineering, University of Waterloo, Waterloo, ON N2L 3G1, Canada

**Keywords:** single microwave photon detection, nitrogen-vacancy centers, dipole-induced transparency, Monte Carlo simulations

## Abstract

We propose a scheme for detecting single microwave photons using dipole-induced transparency (DIT) in an optical cavity resonantly coupled to a spin-selective transition of a negatively charged nitrogen-vacancy (NV^−^) defect in diamond crystal lattices. In this scheme, the microwave photons control the interaction of the optical cavity with the NV^−^ center by addressing the spin state of the defect. The spin, in turn, is measured with high fidelity by counting the number of reflected photons when the cavity is probed by resonant laser light. To evaluate the performance of the proposed scheme, we derive the governing master equation and solve it through both direct integration and the Monte Carlo approach. Using these numerical simulations, we then investigate the effects of different parameters on the detection performance and find their corresponding optimized values. Our results indicate that detection efficiencies approaching 90% and fidelities exceeding 90% could be achieved when using realistic optical and microwave cavity parameters.

## 1. Introduction

As a prerequisite to a broad range of quantum technologies, the realization of highly efficient single-photon detectors has been an interesting research topic for a long time and since the emergence of quantum optics. The detection of microwave photons, which, due to their ultra-small quanta of energy, are typically overwhelmed by the background thermal noise, is a further challenge compared to their optical counterparts. Additionally, there has been a growing demand in recent years for high-fidelity single-photon detectors, which are able to meet the minimum requirements put forward by quantum information processing and quantum cryptography applications. This necessitates detectors with very low dark count rates and an efficiency of close to 100%. In the optical regime, photon detectors of various types, including avalanche photodiodes (APDs), photomultiplier tubes (PMTs), and superconducting nanowire single-photon detectors (SNSPDs), are commercially available and commonly used in experimental setups for applications ranging from fluorescence spectroscopy [1] to quantum key distribution [2]. However, these detectors are limited to operating in the frequency range of hundreds of terahertz and leave the detection of microwave photons as a remaining challenge. As a result, microwave detectors are increasingly desired, as they find applications in circuit quantum electrodynamic [3] and hybrid quantum information processing systems [4], searching for dark matter axions that require detection techniques in the 5 to 500 GHz range [5], and quantum radar [6].

The main challenge with the efficient detection of microwave photons stems from the ultra-small amount of energy quanta in the microwave domain, which can be up to five orders of magnitude smaller than the energy of an optical photon. Consequently, the efficient amplification of single microwave photons, whose energy is comparable to the background noise, even at low temperatures, is impossible with linear amplifiers [7]. In order to fill in this gap, several nonlinear proposals for detection in the sub-terahertz domain have been put forth by researchers in recent years. These proposed detectors can be broadly divided into three general categories, including superconducting detectors, opto-electromechanical detectors, and quantum dot detectors. Superconducting circuits can be regarded as the most mature platform for photon detection in microwave regimes. Most microwave photon detectors experimentally tested, according to the literature, belong to this category. There are proposals based on current-biased Josephson junctions (CBJJ), which can detect propagating microwave photons [8]. The incoming photon, in these detectors, switches the junction from the superconducting state to the resistive state, resulting in a voltage drop across the junction that can then be classically measured. Since the excitation is lost during the detection process, this detection scheme is considered destructive. Furthermore, superconducting qubits have been successfully employed for the quantum nondemolition (QND) measurement of microwave signals resulting in the non-destructive detection of incoming photons. These schemes mostly comprise a superconducting transmon as a three-level quantum system in a transmission line [9]. The microwave photon excites the transmon to an intermediate energy level, triggering its interaction with the probe field. The output field is then monitored by a homodyne detector, based on which the presence of a microwave photon is inferred. Using a flux qubit that is dispersively coupled to a coplanar waveguide (CPW), a detection efficiency of 66% and reset time of 400 ns have been experimentally attained in recent work [10]. By increasing the number of superconducting qubits in the detector design, measurement fidelity can be increased to over 90% [11].

In opto-electromechanical devices, detection is achieved by photon transduction from the microwave to the optical domain, mediated by a mechanical resonator, which induces a strong coupling between different resonating fields [12]. The coupling is based on the interaction of microwave and optical photons with phononic modes of the mechanical resonator through radiation pressure. In order to have a strong coupling between different modes, the mechanical–thermal noise needs to be minimized in this type of detector [13]. Calculated performance parameters render these devices a potential candidate for microwave detection; however, they have not been tested experimentally and proven yet. There are also proposals for semiconductor detectors based on double quantum dots (DQDs) coupled to high-quality factor resonators [14]. One significant advantage of using DQDs in this application is their high tunability through external gate potentials, which makes photon detection in a broad range of frequencies possible. In these sensors, the absorption of microwave photons excites the transition of electrons between the DQDs’ energy levels, which, in turn, results in a change in conductivity. To measure the conductivity, fast charge sensors are needed, which can be realized by capacitively coupling the quantum point contacts (QPCs) to the quantum dot. This type of detector can offer coherence and relaxation times shorter than the detectors based on superconducting qubits [15].

At the same time, solid-state artificial atoms have attracted a large interest among various qubit implementations due to their potential for high scalability and easy integration with electronic circuits. One leading platform of this category is the nitrogen-vacancy (NV) center, a point defect in diamond lattice [16]. Long coherence times, even at room temperature, together with all-optical spin initialization, control and readout capability have made the NV center a promising candidate for many quantum technology applications, including quantum information [17], quantum metrology [18], and quantum spectroscopy [19]. In this article, we introduce a detection scheme for microwave photons based on negatively charged NV centers coupled to optical and microwave cavities. The optical cavity is coupled to a spin-selective transition of the defect, while the electronic spin itself interacts with the microwave cavity field. In this way, the presence of a photon in the microwave cavity can be detected by probing the change in transmissivity of the optical cavity due to the dipole-induced transparency (DIT) effect. We conduct a proof-of-principle simulation study based on a five-energy-level model for the NV center and use the simulation results to investigate the performance of the device under realistic conditions that include imperfect optical photon detection efficiency. We study the effects of different system parameters on the detector’s figures of merit, such as fidelity and efficiency, and extract the optimized values of these parameters under practical limitations.

## 2. Theoretical Model

Nitrogen-vacancy (NV) centers are point defects in diamond crystal lattice, which occur when a nitrogen atom substitutes for a carbon atom and the neighboring site is left empty. They can be realized either during the crystal growth process by introducing nitrogen gas into the chamber or through post-growth methods, such as ion implantation and electron-beam irradiation [20]. There are two charge states of NV centers, neutral (NV^0^) and negative (NV^−^), where the latter exhibits unique spin-dependent photodynamics and will be referred to simply as the NV center in this paper. In the energy level structure predicted by a six-electron model of the NV center, there are two ground and excited spin-triplet states and one metastable singlet state. With no externally applied magnetic field, the spin states $m_{s}=\pm 1$ are degenerate and separated from $m_{s}=0$ with a zero-field splitting at 2.87 GHz [21]. Moreover, as an important feature, the transition between the ground and excited triplets in an NV center is highly spin-preserving and spin mixing occurs mostly when the system decays through the metastable state.

**Figure 1:**
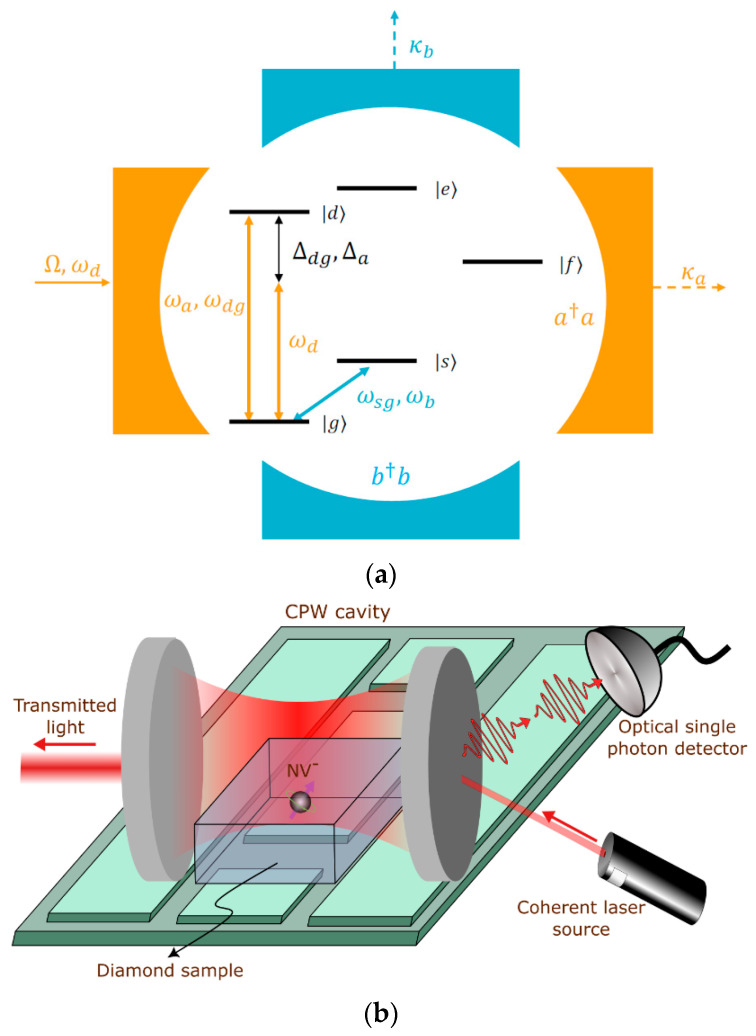
(**a**) Proposed scheme for the detector with a five-level NV center coupled to two cavities; (**b**) illustration of a potential realization of the proposed device using coplanar waveguide (CPW) and a Fabry–Perot cavity.

Figure 1 shows the schematics of the proposed detector comprising an NV center coupled to two cavities: one at an optical frequency (orange) and the other one at a microwave frequency (blue). A five-energy-level model is used for the NV center where |g〉 (|s〉) and |d〉 (|e〉) are the ground and excited triplets with spin $m_{s}=0$ (+1), respectively, and |f〉 is the metastable singlet state. The optical cavity is coupled to the |g〉↔|d〉 transition, as it has only 1% decay through the metastable state, as compared to the 38% from the $m_{s}=+1$ transition (the |s〉 to |e〉 transition). That is because, in the former case, the NV center interacts longer with the optical cavity before the population accumulates in the metastable state. This results in a higher number of blocked photons and, consequently, a higher contrast of the detector according to the detection scheme explained below. At the same time, photons in the microwave cavity resonate with the electronic spin of the ground states by exciting the |g〉↔|s〉 transition. The transmissivity of the optical cavity is measured by illuminating the cavity with a coherent probe laser of frequency $\omega_{d}$ and drive strength Ω, determined by the power of the incident laser and its coupling efficiency into the cavity. Because of the DIT phenomenon [22], the measured transmissivity of the optical cavity now depends on whether the cavity interacts with the NV center inside of it or not, which, in turn, is a function of the NV spin state. Consider an NV center initially in state |g〉. If no microwave photon is present, the NV center will remain in the |g〉 state. This will cause the cavity spectrum to split, which will block the transmission of resonant photons through the cavity. When a microwave photon is present, the NV spin switches, exciting the NV into the |s〉 state, which has a much weaker coupling to the cavity. The optical cavity will appear empty and resonant photons will be transmitted. Thus, by measuring either the blocked or transmitted optical photons, we can detect a single microwave photon.

Before further discussion, it is worth mentioning that a similar scheme, including NV centers coupled to two cavity modes, has been investigated in the literature for microwave-to-optical transduction in the quantum regime [23,24]. The proposed idea relies on the NV centers to enable a Raman-based coherent transfer of quantum states between different cavities within the system. While these devices can be efficiently used to interconnect distant quantum computers or for qubit storage and retrieval, they may be seen as over-engineered for the specific application of photon detection, where the qubit decoherence is not of concern. Compared to those works, the present scheme in this paper enjoys more simplicity in the design and fabrication, as it has no need for external control beams or accurate magnetic fields. Additionally, as will be evidenced by the results, the DIT process employed in this study leads to a lower sensitivity of the device to an imperfect efficiency of the optical detector and coherence properties of the system. The total Hamiltonian of the system in a rotating frame can be written as follows:

(1) $$\begin{array}{l} H_{0}/\hbar =\Delta_{dg}|d\rangle \langle d|+\Delta_{e}|e\rangle \langle e|+\Delta_{a}\left(a^{†}a\right)+\Delta_{b}\left(b^{†}b\right)\\ H_{\mathrm{int}}/\hbar =g_{a}\left(a|e\rangle \langle s|+a^{†}|s\rangle \langle e|\right)+g_{a}\left(a|d\rangle \langle g|+a^{†}|g\rangle \langle d|\right)+g_{b}\left(b|s\rangle \langle g|+b^{†}|g\rangle \langle s|\right)\\ H_{\mathrm{drive}}/\hbar =\Omega \left(a^{†}+a\right)\\ H=H_{0}+H_{\mathrm{int}}+H_{\mathrm{drive}} \end{array}$$

where it has been transformed using the following rotating frame unitary:

(2) $$U=\exp\left(it\left[\omega_{d}\left(a^{†}a+|d\rangle \langle d|\right)+\left(\omega_{sg}+\omega_{d}\right)\left(|e\rangle \langle e|\right)+\omega_{sg}\left(|s\rangle \langle s|+b^{†}b\right)\right]\right)$$

As can be seen in this equation, the total interaction Hamiltonian is the sum of the interaction energies between the quantum system and different cavity fields. A similar scenario can also occur in other systems, where the quantum emitter is coupled to multiple cavity modes, such as bimodal cavities [25,26,27,28,29] or magnonic systems [30,31,32]. The detuning parameters used in Equation (1) are defined as $\Delta_{dg}=\omega_{dg}-\omega_{d}$, $\Delta_{e}=\omega_{e}-\omega_{sg}-\omega_{d}$, $\Delta_{a}=\omega_{a}-\omega_{d}$, and $\Delta_{b}=\omega_{b}-\omega_{sg}$. In the notation used, a and b are photon annihilation operators of the optical and microwave cavities, respectively. Moreover, $\omega_{a}$, $\omega_{b}$, $g_{a}$, $g_{b}$, $\kappa_{a}$, and $\kappa_{b}$ are their corresponding resonance frequencies, coupling constants, and decay rates.

It is worth noting that the Hamiltonian in Equation (1) is essentially composed of three Jaynes–Cummings Hamiltonians, where each can be proved to conserve the angular momentum in the z-direction for the case of a spherically symmetric atom. However, this may be violated in our case, where there are multiple transitions of a single emitter coupled simultaneously to the cavity system, and, additionally, the emitter is an NV center with a trigonal pyramidal (C_3V_) symmetry instead of a spherical one. To investigate it in more detail, we can consider transitions coupled to the optical and microwave cavities separately. Regarding the optical domain transitions, we know that they are highly spin-conserving in the NV centers [33] and, thus, are activated by unpolarized photons inside the optical cavity. Therefore, provided that the effect of this trigonal symmetry is brought into account in the coupling factors of different transitions, considering the orientation of the NV axis with respect to the cavity field, the Hamiltonian can predict the dynamics of the emitter-cavity system and conserves the angular momentum at the same time. However, in the case of spin-flipping microwave transitions between different ground states, depending on the orientation of the NV axis with respect to both the cavity field and the crystal lattice, the angular momentum conservation may be violated when the phononic interaction inside the solid-state material is neglected. In this study, the results are presented for a broad range of cavity parameters that agree with previous experimental works. Therefore, while these phononic interactions can affect the agreement of experimental measurement and theoretically predicted coupling parameters under different excitation conditions, they do not undermine the overall validity of the results presented in this paper. As an open quantum system, the dynamics of the device can be modeled using a Lindblad master equation, which governs the evolution of the system density matrix ρ and is of the following form [34]:

(3) $$\frac{d\rho \left(t\right)}{dt}=-\frac{i}{\hbar }\left[H,\rho \left(t\right)\right]+\sum_{k}\frac{1}{2}\left[2C_{k}\rho \left(t\right)C_{k}^{†}-\rho \left(t\right)C_{k}^{†}C_{k}-C_{k}^{†}C_{k}\rho \left(t\right)\right]$$

where the $C_{k}$’s are standard collapse operators, which, in this case, bring into account the population decay from both the excited and metastable states, pure dephasing of the excited states, and cavity decay:

(4) $$\begin{array}{cc} C_{1}=\sqrt{\left(0.99\right)\left(0.99\right)\Gamma_{d}}|g\rangle \langle d| & C_{2}=\sqrt{\left(0.01\right)\left(0.99\right)\Gamma_{d}}|s\rangle \langle d|\\ C_{3}=\sqrt{\left(0.01\right)\Gamma_{d}}|f\rangle \langle d| & C_{4}=\sqrt{\left(0.99\right)\left(0.46\right)\Gamma_{e}}|s\rangle \langle e|\\ C_{5}=\sqrt{\left(0.99\right)\left(0.46\right)\Gamma_{e}}|g\rangle \langle e| & C_{6}=\sqrt{\left(0.54\right)\Gamma_{e}}|f\rangle \langle e|\\ C_{7}=\sqrt{\left(0.99\right)\Gamma_{f}}|g\rangle \langle f| & C_{8}=\sqrt{\left(0.01\right)\Gamma_{f}}|s\rangle \langle f|\\ C_{9}=\sqrt{\gamma }|d\rangle \langle d| & C_{10}=\sqrt{\gamma }|e\rangle \langle e|\\ C_{11}=\sqrt{\kappa_{a}}a & C_{12}=\sqrt{\kappa_{b}}b \end{array}$$

where $\Gamma_{x}$ is the total population decay rate from state |x〉 and γ stands for the pure dephasing rate of the excited states. The numeric coefficients appearing in Equation (4) are branching ratios that denote the probability of decay through each of the available quantum channels. When using these branching ratios, it would be possible to define a separate decay rate and, consequently, a separate collapse operator responsible for the system jump through each decay channel. Therefore, $C_{1}$, $C_{2}$, and $C_{3}$ account for the decay from |d〉 with a 1% chance of direct intersystem crossing to |s〉 and a 1% chance of decaying to the metastable state. Similarly, $C_{4}$, $C_{5}$, and $C_{6}$ account for the decay from |e〉. Additionally, $C_{7}$ and $C_{8}$ represent the decays out of the shelving state and $C_{9}$ and $C_{10}$ are the dephasing of the excited states. Moreover, the photons’ decay out of the cavities is accounted for by $C_{11}$ and $C_{12}$. It should be noted, however, that the amount of intersystem crossing and decay to the shelving state depends on the applied axial magnetic field $B_{z}$, and the above values are for the case of $B_{z}=0$ [35].

To obtain the results presented in the next section, we simulated the dynamics of the proposed scheme by two different approaches, both implemented with the QuTiP package in Python. The first approach uses a direct numerical integration of the governing Lindblad master equation for the Hamiltonian introduced in Equation (1). The second uses the quantum trajectory approach based on the Monte Carlo wave function (MCWF) method. The quantum jump or Monte Carlo wave function method is a member of a broad family of stochastic techniques known as the quantum Monte Carlo (QMC) methods, widely used in simulations of open quantum systems. The idea behind this strategy is to simulate the time evolution of the wave function of a quantum system by using a statistical sampling approach [36]. It is based on the interpretation of the wave function as a probability amplitude and uses random sampling to estimate the time-dependent expectation values of the observables of interest. In the MCWF simulation, the Lindblad master equation of the system is divided into two parts, where the first part involves using a non-Hermitian Hamiltonian to evolve the state vector of the system and calculate the probability of a quantum jump at each step.The second part of the master equation includes one or more collapse operators responsible for the interaction of the system with the environment and is applied to the state vector whenever a jump occurs. Finally, the quantum state of the system can be obtained by averaging over many of these calculated trajectories. It should be noted that, although the MCWF method offers advantages for simulating large and complex quantum systems, it is subject to certain assumptions and limitations. In particular, the system is assumed to be weakly coupled to the environment so that the dynamics of the system can be treated perturbatively, and it becomes computationally expensive as the number of the quantum states of the system increases.

## 3. Results and Discussion

To obtain a realistic evaluation of the proposed detector scheme, we use cavity parameters that have been experimentally realized [37,38,39,40,41,42,43] or theoretically proposed [44] in the existing literature. For the optical cavity, the cooperativity η is related to the Purcell enhancement P by [45]:

(5) $$\eta =\frac{4g^{2}}{\kappa \Gamma }=P\left(\frac{\Gamma_{ZPL}}{\Gamma +\gamma }\right)$$

where Γ is the total longitudinal decay, $\Gamma_{ZPL}$ is the decay into the zero-phonon line, and γ is the dephasing rate. In terms of the longitudinal and coherence lifetimes, τ and $\tau^{*}$, the cooperativity is:

(6) $$\eta =P\left(\frac{\varepsilon /\tau }{1/\tau^{*}}\right)$$

where ε is the Debye–Waller factor, which is 0.03 for the NV centers [45]. Using lifetime values of τ=11.9 ns and $\tau^{*}=5.8\;\mathrm{ns}$ [46], the cooperativity is η=0.015 P. Table 1 shows the Purcell enhancement and corresponding cooperativities for a number of optical cavities reported in the literature.

Based on the values in this table, the cooperativity is η=0.4 with a quality factor of $Q_{a}=10^{6}$ for the optical cavity parameters in most of our simulations. For the microwave cavity coupled to the negatively charged NV centers in the diamond lattice, one feasible option, according to the literature, is a coplanar waveguide (CPW) cavity [23,44,47]. In the hybrid device proposed by Li et al., a microscale diamond beam with a single built-in NV center is coupled to a CPW cavity [44]. The diamond beam couples to the cavity photons via a dielectric interaction, and the motion of the beam couples to the spin through a magnetic field gradient. Based on the predicted coupling strength and decay for this cavity, we used a coupling constant of $g_{b}=2\pi \times 10\;\mathrm{kHz}$ and a quality factor of $Q_{b}=10^{6}$. Moreover, the transition frequencies and decay rates of the NV center used in the simulations are given in Table 2. The microwave and the optical cavities are assumed to be in resonance with their respective transitions between the levels of the NV center.

After the microwave photon enters the cavity, it takes half a Rabi cycle ($\tau_{1/2}=\pi /\left(2g_{b}\right)$, where $2g_{b}$ is the single-photon Rabi frequency) to excite the NV center into the |s〉 state. The microwave cavity decay rate, $\kappa_{b}$, and the microwave cavity coupling constant, $g_{b}$, determine the population of the |s〉 state at time $\tau_{1/2}$. Since the transmissivity of the cavity depends on the |g〉 state population, the performance of the detector is dominantly affected by the microwave cavity parameters. The system experiences dissipation and decays as $\exp[-(\Gamma_{sg}+\kappa_{b})t/2]$. Thus, we expect the population of |s〉 after half a Rabi cycle to be:

(7) $$\rho_{ss}=\exp\left[-\left(\Gamma_{sg}+\kappa_{b}\right)\pi /4g_{b}\right]$$

**Figure 2:**
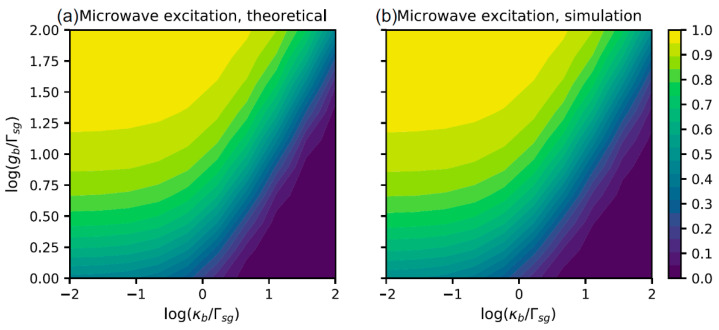
Dependence of state |s〉 population on the cavity decay rate and coupling constant; (**a**) theoretical result, based on Equation (7); (**b**) simulation result, using the scheme in Figure 1.

The dependence of $\rho_{ss}$ on both $g_{b}$ and the microwave cavity decay rate $\kappa_{b}$ is shown in Figure 2.

At $t=\tau_{1/2}$, a probe laser resonance with the optical cavity ($\Delta_{a}=0$) probes it with a drive strength of $\Omega_{d}=0.3g_{b}$. This provides an expectation value of 0.001 optical cavity photons in a steady state, minimizing the nonlinear effects that can undesirably affect the performance. With this drive strength, the rate of transmitted photons through an empty cavity is $<2\times 10^{6}$ photons/second; therefore, we do not expect to see the saturation of an optical single-photon detector, such as a single-photon avalanche photodiode (SPAD) or a superconducting nanowire single-photon detector (SNSPD).

**Figure 3:**
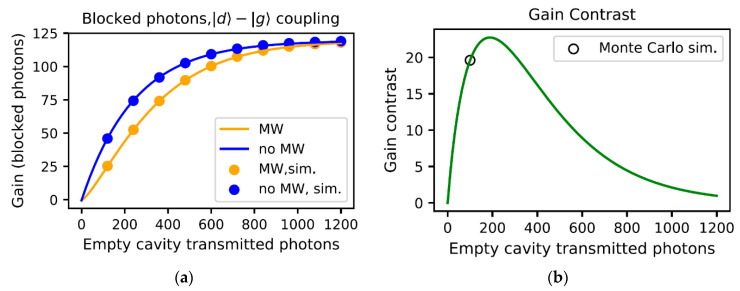
(**a**) Detector gain when a microwave photon is not present (blue) and when a microwave photon is present (orange). The dots are the results of the Monte Carlo simulation, and the solid lines are obtained by direct numerical integration of the master equation. (**b**) Gain contrast. The black circle shows the gain contrast for 100 photons obtained by the Monte Carlo simulation.

Figure 3a shows the gain of the detector, defined as the number of blocked optical photons as a function of the total number of illuminated photons or, equivalently, the number of transmitted photons if the cavity was empty. As can be seen in this figure, when there is no microwave photon, and the NV center is in the |g〉 state, it will block the photons up to a saturation point. The saturation occurs as the population accumulates in the shelving state with a considerably longer lifetime. As a microwave photon enters the cavity, some of the population is excited into the |s〉 state and no longer interacts with the optical cavity, resulting in a smaller number of blocked photons. As time goes on, the NV center will be de-excited into the ground state, where it will block subsequent photons. Moreover, due to this decay of the |s〉 state population, both cases, with and without microwave photons, converge to an almost equal saturation level. The predicted gain plotted in this figure is based on the scattering and transmission probabilities, which on resonance are $2\eta /{\left(1+\eta \right)}^{2}$ and $1/{\left(1+\eta \right)}^{2}$, respectively, while bringing into account the exponential decay of the |s〉 state population. The gain contrast is the difference between the two plots in Figure 3a, allowing us to choose an appropriate number of incident photons. The gain contrast is plotted in Figure 3b. The result of the Monte Carlo simulation for 100 incident photons is also presented in this figure and agrees well with the results from the master equation integration approach. This number of incident photons provides a high gain contrast with a reasonable simulation time.

To use this system as a microwave photon detector, we set a threshold value t for the number of detected photons. If we detect more than t photons, we can say a microwave photon exists. The detection fidelity

(8) $$F=1-\frac{1}{2}\left(\varepsilon_{0}+\varepsilon_{1}\right)$$

quantifies the performance of the detector, where $\varepsilon_{0}$ and $\varepsilon_{1}$ are the false negative and false positive rates, respectively. The detection efficiency is the number of microwave photons successfully detected, corresponding to the true positive rate. These two parameters, together with the signal-to-noise ratio (SNR), can be used to evaluate the performance of a detector and are studied to investigate the performance of the proposed scheme. In this single-photon detector based on an NV center in a microwave cavity, assuming that the microwave photon can successfully reverse the NV spin and excite the whole population to the |s〉 state, the fidelity of the detector will be the same as that of the spin readout technique used [48]. For the proposed scheme in this paper, if we assume a cooperativity of 0.4 and a perfect optical detector efficiency, the fidelity is F=99.6% or SNR=11.31.

**Figure 4:**
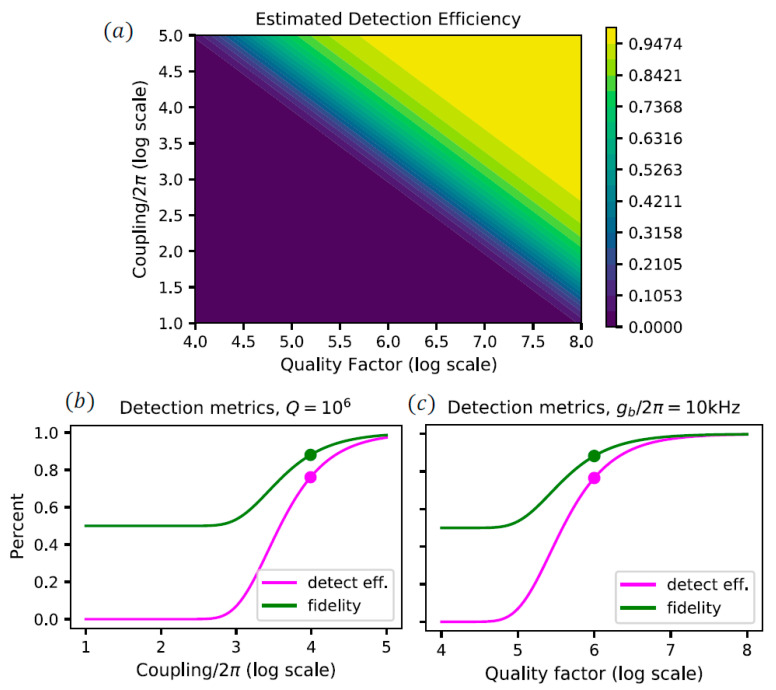
Effects of the coupling, $g_{b}$, and quality factor, $Q_{b}$, of the microwave cavity: (**a**) Estimated detection efficiency for a range of microwave cavity couplings and quality factors, (**b**) estimated detection efficiency and measurement fidelity for a constant quality factor $Q_{b}=10^{6}$. (**c**) Same as (**b**), but now for a constant coupling $g_{b}/2\pi =10\;\mathrm{kHz}$. The dots represent the microwave cavity parameters proposed in Ref. [44]. The parameters of the optical cavity are η=0.4 and $Q_{a}=10^{6}$ in these simulations.

To account for the effect of a non-ideal microwave cavity on the detector performance, we can obtain a rough estimate of the predicted detection by looking at the probability that the NV center is excited into the |s〉 state. We assume that the threshold value is equal to the incident photon number (i.e., if 25 photons are injected and all 25 photons are transmitted, one can conclude that a microwave photon is present). To estimate the readout fidelity, we assume that the false positive rate is 0 and the false negative rate is the probability that a microwave excitation fails. The estimates are shown in Figure 4.

One potential solution for further enhancement of the above-calculated efficiency is to use an ensemble of NV centers instead of a single NV. In this case, the effective cooperativity scales as HN, where N is the number of emitters and H is a collective coupling parameter that describes the ordering of the system. If all emitters are at the antinode of the standing wave, H=1. If they are randomly distributed, 〈H〉=1/2. Assuming that H=1, the effective cooperativity is:

(9) $$\eta_{eff}=N\eta$$

where η is the single emitter cooperativity. Moreover, since the coupling constant relates to the cooperativity according to Equation (5), the effective coupling constant is given by:

(10) $$g_{eff}=\sqrt{N}g$$

**Figure 5:**
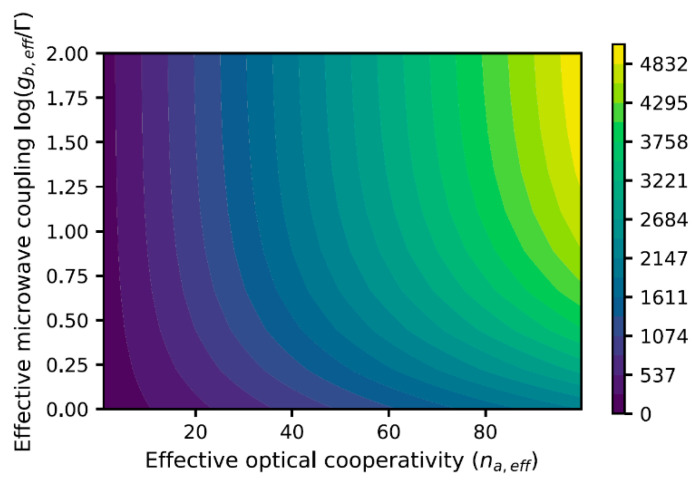
Saturation gain as a function of optical cavity cooperativity and microwave cavity coupling strength in a detector comprising an ensemble of NV centers.

Using an ensemble of emitters will affect our results in two ways. First, by enhancing the microwave cavity coupling, $g_{b,\mathrm{eff}}=\sqrt{N}g_{b}$, it will improve the population transfer to |s〉. Second, by enhancing the optical cavity cooperativity, it reduces cavity transmission and scattering. On resonance, the transmission and scattering probabilities are $T=1/{\left(1+N\eta_{a}\right)}^{2}$ and $T=2N\eta_{a}/{\left(1+N\eta_{a}\right)}^{2}$, respectively. Figure 5 shows how the gain saturation depends on the effective optical cavity cooperativity and effective microwave cavity coupling constant.

It should be noted that there are two main challenges when working with an ensemble for the proposed scheme. First, the increased coupling in the optical cavity may reduce the gain contrast. Second, inhomogeneous broadening will affect the coupling and transmission spectra. To elaborate on the former challenge, as we know, cavity–atom coupling will cause a dip in the transmission spectrum. As the coupling increases, the dip becomes wider. Consequently, while higher cooperativities result in a system more robust to frequency changes, it can lead to a reduction of the gain contrast. Regarding the second challenge, according to the work of Diniz et al., the dip in the transmission spectrum for a cavity coupled to an ensemble of NV centers disappears as the width of the broadening exceeds the coupling strength [49]. Therefore, the appropriate number of NV centers in the ensemble must be chosen based on a trade-off between inhomogeneous broadening and cooperativity enhancement.

**Figure 6:**
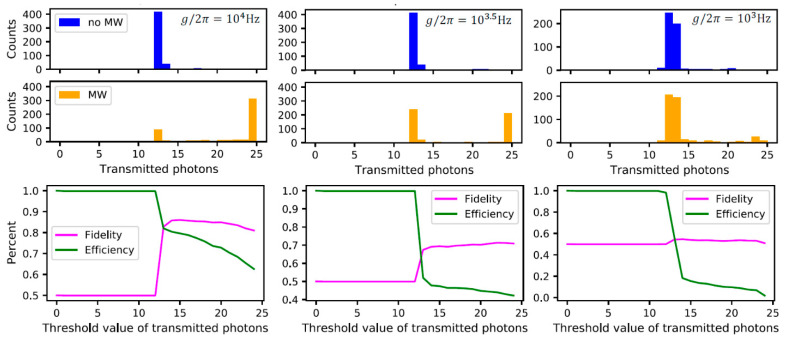
Top row: Results of Monte Carlo simulations. The plots show the number of transmitted photons when 25 photons are incidents on the cavity. The counts correspond to the number of Monte Carlo trajectories. Bottom row: The detection efficiency and fidelity for different choices of the threshold value. When the number of detected photons is above the threshold value, we conclude that a microwave photon is present. The microwave cavity coupling decreases from left to right.

To see how the coupling constant of the microwave cavity affects the photon count statistics and, in consequence, the fidelity and efficiency of the detector, a Monte Carlo simulation was performed with 500 trajectories. Taking the quality factor to be $Q_{b}=10^{6}$, we can see from Figure 4b that the lower bound on the coupling strength is $g_{b}/2\pi =10^{3}\;\mathrm{Hz}$, which provides a detection efficiency of around 10% and a fidelity just larger than 50%. To achieve a fidelity of at least 70%, we require the coupling strength to be $g_{b}/2\pi =10^{3.5}\;\mathrm{Hz}$. The Monte Carlo simulations for these two values of $g_{b}$, as well as the theoretically achievable value of $g_{b}/2\pi =10^{4}\;\mathrm{Hz}$, are shown in Figure 6.

**Figure 7:**
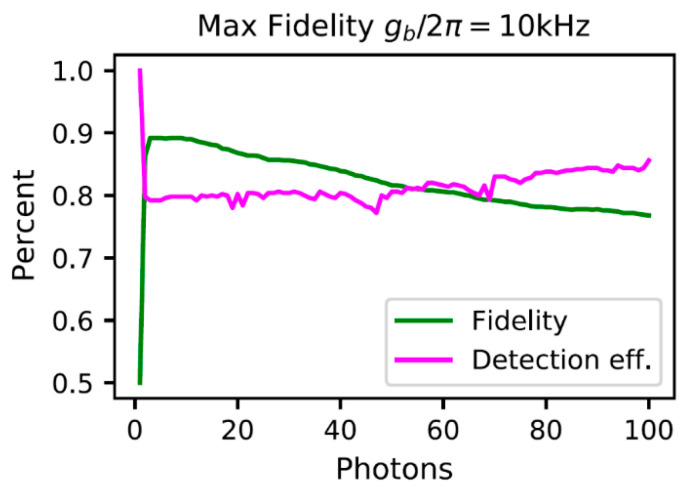
The maximum achievable fidelity and corresponding efficiency vs. number of incident photons.

We can also look at how the number of incident photons will affect the measurement fidelity since the more photons interacting with the NV center, the more likely it will scatter out of the ground state. The maximum fidelity is shown in Figure 7, for $g_{b}/2\pi =10\;\mathrm{kHz}$ and $Q_{b}=10^{6}$. As can be seen in this figure, these microwave cavity parameters would allow a fidelity of up to 90% to be achieved with the proposed scheme.

**Figure 8:**
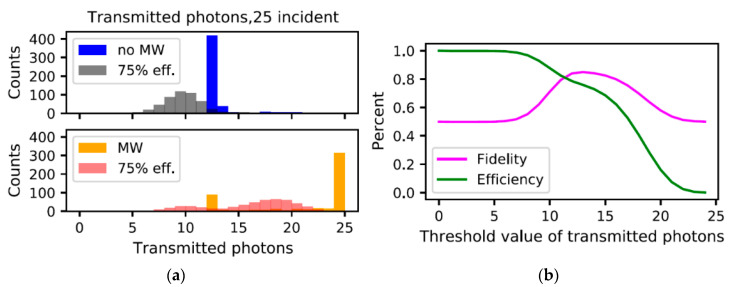
(**a**) Adjusted histograms, accounting for imperfect optical photon detector. The microwave parameters are $g_{b}/2\pi =10\;\mathrm{kHz}$ and $Q_{b}=10^{6}$. (**b**) Fidelity and microwave detection efficiency for the adjusted histograms.

Lastly, we investigate the effects of imperfect optical photon detection on the performance of our detector. Figure 8a shows the adjusted histograms once we account for a detector with 75% efficiency, which is slightly better than SPAD (~70% at 650 nm) and sits comfortably within the performance of SNSPDs. The microwave detection fidelity and efficiency are also plotted in Figure 8b. As can be seen in this figure, for smaller numbers of incident photons, the detection efficiency will affect the measurement fidelity, as the histograms have more overlap.

## 4. Conclusions

To summarize, we presented a scheme for detecting single microwave photons using a solid-state platform based on NV centers in diamond lattice simultaneously coupled to two cavities—one optical and one microwave. The scheme employs dipole-induced transparency (DIT) to optically read out the NV spin and consequently detect the presence of microwave photons interacting with it. A Monte Carlo wave function (MCWF) analysis was carried out, together with numerical integration of the governing Lindblad master equation, to simulate the system’s dynamics. The simulations were based on the realistic cavity parameters from the existing literature and tried to account for some potential experimental imperfections. Measurement-related parameters, such as the number of incident photons and gain threshold, were optimized using the obtained results. The findings suggest that the proposed scheme can be potentially used to realize practically useful solid-state detectors of single microwave photons operating with high fidelity and efficiency, as required in quantum information processing and cryptography, as well as in quantum sensing applications.

## Figures and Tables

**Table 1:** Optical cavities for NV centers and their estimated cooperativities and quality factors.

Author	Resonator Type	Quality Factor	Purcell Enhancement	Estimated Cooperativity
Faraon et al. [37]	Ring resonator	$4\times 10^{3}$	10	0.15
Faraon et al. [38]	PCC	$3\times 10^{3}$	70	1.05
Janitz et al. [39]	Fabry Perot	$1\times 10^{6}$	20	0.3
Hausmann et al. [40]	Nanobeam	$6\times 10^{3}$	7 (expected 34)	0.105 (0.51)
Lee et al. [41]	Nanobeam	$2\times 10^{4}$	26	0.364
Gould et al. [42]	PCC, GaP	$8\times 10^{3}$	16	0.24
Barclay et al. [43]	Ring resonator	$3\times 10^{3}$	10	0.25

**Table 2:** NV center parameters as used in our simulations.

Parameter	Values
Optical transition, $\omega_{dg}$	2π×470 THz
Microwave transition, $\omega_{sg}$	2π×3.4 GHz
|d〉 $\mathrm{decay},\Gamma_{d}$	1/(12 ns)
|e〉 $\mathrm{decay},\Gamma_{e}$	1/(7.5 ns)
|s〉 $\mathrm{decay},\Gamma_{s}$	2π×21.2 Hz

## Data Availability

Not applicable.
